# How accurately are subthalamic nucleus electrodes implanted relative to the ideal stimulation location for Parkinson’s disease?

**DOI:** 10.1371/journal.pone.0254504

**Published:** 2021-07-15

**Authors:** Patrick Pearce, Kristian Bulluss, San San Xu, Boaz Kim, Marko Milicevic, Thushara Perera, Wesley Thevathasan

**Affiliations:** 1 Bionics Institute, East Melbourne, Victoria, Australia; 2 Department of Neurosurgery, St Vincent’s Hospital Melbourne, Fitzroy, Victoria, Australia; 3 Department of Neurosurgery, Austin Hospital, Heidelberg, Victoria, Australia; 4 Department of Surgery, The University of Melbourne, Parkville, Victoria, Australia; 5 Medical Bionics Department, The University of Melbourne, East Melbourne, Victoria, Australia; 6 Department of Neurology, Austin Hospital, Heidelberg, Victoria, Australia; 7 Department of Medicine, The University of Melbourne, Parkville, Victoria, Australia; 8 Department of Neurology, The Royal Melbourne Hospital, Parkville, Victoria, Australia; Oslo Universitetssykehus, NORWAY

## Abstract

**Introduction:**

The efficacy of subthalamic nucleus (STN) deep brain stimulation (DBS) in Parkinson’s disease (PD) depends on how closely electrodes are implanted relative to an individual’s *ideal* stimulation location. Yet, previous studies have assessed how closely electrodes are implanted relative to the *planned* location, after homogenizing data to a reference. Thus here, we measured how accurately electrodes are implanted relative to an ideal, dorsal STN stimulation location, assessed on each individual’s native imaging. This measure captures not only the technical error of stereotactic implantation but also constraints imposed by planning a suitable trajectory.

**Methods:**

This cross-sectional study assessed 226 electrodes in 113 consecutive PD patients implanted with bilateral STN-DBS by experienced clinicians utilizing awake, microelectrode guided, surgery. The error (Euclidean distance) between the actual electrode trajectory versus a nominated ideal, dorsal STN stimulation location was determined in each hemisphere on native imaging and predictive factors sought.

**Results:**

The median electrode location error was 1.62 mm (IQR = 1.23 mm). This error exceeded 3 mm in 28/226 electrodes (12.4%). Location error did not differ between hemispheres implanted first or second, suggesting brain shift was minimised. Location error did not differ between electrodes positioned with (48/226), or without, a preceding microelectrode trajectory shift (suggesting such shifts were beneficial). There was no relationship between location error and case order, arguing against a learning effect.

**Discussion/Conclusion:**

The proximity of STN-DBS electrodes to a nominated ideal, dorsal STN, stimulation location is highly variable, even when implanted by experienced clinicians with brain shift minimized, and without evidence of a learning effect. Using this measure, we found that assessments on awake patients (microelectrode recordings and clinical examination) likely yielded beneficial intraoperative decisions to improve positioning. In many patients the error is likely to have reduced therapeutic efficacy. More accurate methods to implant STN-DBS electrodes relative to the ideal stimulation location are needed.

## Introduction

A successful therapeutic outcome from deep brain stimulation (DBS) requires fulfilment of three crucial steps–appropriate patient selection, electrodes accurately implanted with close proximity to the ideal stimulation location, and good programming [1]. Of these, electrode location is the easiest to objectively measure [2]. Hitherto, most studies have focussed on measuring how closely electrodes are implanted relative to the *planned* location [3–5]. This measure provides insights into the technical constraints of stereotactic neurosurgery [6, 7]. However, it is proximity to the *ideal* location, the ‘sweet spot’ where stimulation can achieve the maximal benefit, that determines efficacy [8, 9]. Such proximity to the ideal location depends not only on the technical error of stereotactic implantation but also constraints imposed by planning a suitable trajectory [10–12]. Trajectory plans need to accommodate for individual differences in anatomy such as cortical shape, ventricular size, blood vessel location and target orientation [13–15]. How the target is traversed is also important, preferably offering some redundancy within a beneficial zone if the single ideal stimulation location is missed [16]. Many different approaches can be taken in the same brain, and ultimately planning involves heuristic decisions that vary between, and even within, clinicians [17].

Currently, the predominant DBS procedure for Parkinson’s disease (PD) involves implantation of electrodes into the subthalamic nucleus (STN), often aided by microelectrode recordings and clinical testing on awake patients [18]. Yet, there is insufficient information on how accurately such electrodes are implanted, relative to the ideal location. One limiting factor has been controversy regarding the ideal anatomical location to apply DBS in the subthalamic region [17]. A converging body of evidence now suggests that, on average, this ‘sweet spot’ is in the dorsal STN region just below the superior margin of the adjacent red nucleus [8, 9, 19–22]. Moreover, many previous studies assessing electrode location error have suffered the limitation of taking individual data and deforming into a common imaging space or referencing to a common landmark such as the midcommissural point [3, 19, 23, 24]. Such methods introduce a significant confound given that the relative location of the STN varies greatly between individuals [12, 25].

An understanding of how accurately electrodes are implanted relative to the ideal, dorsal STN stimulation location is important, as it can inform on methods that could improve outcomes [9]. For example, previous studies of mixed DBS targets have suggested that the first hemisphere implanted may have a smaller error, suggesting that clinicians should implant the most clinically important side first [26, 27]. Surgical experience is considered an important determinant of DBS outcomes and clinicians are encouraged to accrue an adequate caseload under supervision before embarking on their own DBS practice [28–31]. The exact surgical method to implant STN DBS is also controversial. In particular, it remains contentious as to whether microelectrode recordings and awake surgery with intraoperative clinical assessments facilitate decisions that improve outcomes [18, 32, 33]. Finally, it is unclear how often the discrepancy in actual versus ideal STN DBS electrode location can be corrected by the new generation of electrodes that can ‘steer’ stimulation [34].

Thus here, in a cohort of 226 consecutive electrodes in 113 patients implanted with STN DBS for PD by experienced clinicians utilizing awake microelectrode guided surgery, we assessed the error (measured as Euclidean distance) between the actual electrode trajectory versus a nominated, ideal dorsal STN stimulation location, systematically identified in every individual’s native imaging. Furthermore, we assessed whether this error is predicted by case order (to seek a learning effect) or the order of hemisphere implanted (first versus second side). To inform on whether the technique of microelectrode recordings with clinical assessments on awake patients led to decisions that improved outcomes, we compared the error between electrodes positioned with, or without, a preceding intraoperative trajectory change.

## Materials and methods

St Vincent’s Hospital Melbourne ethics committee approval was obtained (HREC/17/SVHM/81). The Ethics review board determined that patient consent was unnecessary given that only anonymised data was analysed. We retrospectively acquired, and deidentified, imaging and medical records for consecutive patients with PD implanted with STN-DBS by a single neurologist (WT) and neurosurgeon (KB) DBS surgical team from onset of independent DBS practice on 16 November 2013 until study conception on 14 September 2017. Prior to this period, both clinicians had undertaken DBS specific fellowships and experienced over 75 supervised DBS surgeries. Patients with PD were selected for STN-DBS due to motor fluctuations and/or drug refractory tremor. One hundred and sixteen patients with PD who received STN-DBS in the study period were identified from a database. Three patients were excluded due to incomplete data (imaging in two, operative notes in one). Data from revision surgeries were not included.

Pre-operatively, volumetric magnetic resonance imaging (MRI) at 3 tesla including FLAIR and T1 with contrast was acquired in the axial plane with a 1 mm slice thickness. Planning was performed by the neurologist (WT) on a Stealth Surgical Navigation System (Medtronic, Dublin, Ireland), with the STN visualised on FLAIR and other aspects of the trajectory on the contrast enhanced T1 [35]. Typically, the trajectory to the STN involved a ring of 65–73 degrees and an arc of 18–25 degrees, with an entry point anterior to the motor strip, allowing a wide margin from the ventricles and a trajectory oriented towards the axis of the STN, aiming to implant a long span. Planning aimed to achieve a trajectory passing through the ‘ideal’ dorsal STN before reaching a termination point in the ventral STN midway between the medial-lateral extent of the STN at the Bejjani line [16]. In our experience, the issue is not whether a trajectory can be found that reaches the single ideal anatomical location within the dorsal STN but to create a trajectory that also provides; 1) sufficient span/redundancy within the STN to account for stereotactic error (between the actual versus intended trajectory), and 2) accounts for the possibility that, in a given individual, the ideal functional location for DBS may be more ventral than anatomy would suggest. Thus, trajectory plans inevitably entail compromises between these objectives whilst also maximising safe passage by avoiding sulci, ventricles, and visible blood vessels.

Fundamental aspects of the surgical technique to implant STN DBS remained stable over the study period. Bilateral brain electrode implantation was performed with the patient awake (with short acting sedation and analgesia applied intermittently during the burr hole drill and at other times for patient comfort). In the same operative session, the intracranial electrodes were then connected to the extension cable and the pulse generator (Activa RC, Medtronic, Dublin, Ireland) under general anaesthesia. There was a preference to implant the worst affected side first [26, 27]. After placement of a stereotactic frame (CRW; Integra Life-sciences Corporation, Plainsboro, NJ), a contrast-enhanced computed tomography (CT) scan was obtained and fused with the preoperative MRI. The patients head was positioned at 30 degrees. After the Burr-hole, a cannula was inserted either 15 or 20 mm above the ventral STN target (at the Bejjani line) and dural sealant (Tisseal, Baxter, Vienna, Austria) was applied. An initial single trajectory was then explored through the cannula using a microelectrode (FHC, Bowdoin, Maine, USA), with single and multiunit recordings captured using the LeadPoint system (Medtronic, Dublin, Ireland) [36]. Recordings typically commenced 10 mm above the ventral STN target and proceeded towards it. At each 1mm increment, 3 second recordings were saved to the raster–with continuous observation of recordings between these steps. Rest recordings were assessed without specific sensorimotor testing. STN activity was usually observed between around 3–6 mm above and 1 mm below the ventral STN target (i.e. a typical STN span of 4-6mm). Thereafter, microelectrode recordings were continued for another 1–3 mm to seek substantia nigra pars reticulata activity.

After recordings, the recording tip of the microelectrode was retracted and the stimulating tip of the microelectrode assembly advanced to apply DBS to the dorsal STN (typically 1-2mm below STN activity detected by microelectrode recordings) and then, again, 2 mm below that level. Such test stimulation was always applied at 60 μsec and 130 Hz with the cathode being the stimulating ring of the microelectrode assembly and the anode being a retractor attached to the frame. If well placed, clinical benefits on rigidity and akinesia were typically evident at low amplitudes (1–2 milliamps (mA)) and side effects at higher amplitudes (e.g. ≥ 3–4 mA).

If the initial testing suggested inadequate positioning, a new single-track parallel trajectory was explored by removing the microelectrode and cannula and reinserting at a vector selected by the surgical team using the 2 mm increment options available on the star-drive. Based on our own experience, we came to consider the following as relative indications to change trajectory; STN microelectrode recording span < 4 mm, lack of motor benefit with DBS amplitude ≤ 2 mA, medial side effects with DBS amplitude < 4 mA (e.g. ipsilateral dilated pupil, heat sensation, nausea), corticospinal side effects with DBS amplitude < 2.5 mA, and any significant occurrence of affective side effects. When a trajectory was deemed satisfactory, the microelectrode (diameter 0.55 mm) and the inner reducing sleave of the insertion tube (diameter 0.7 mm) were removed, thus allowing passage of the macroelectrode (diameter 1.25 mm, model 3387; Medtronic, Dublin, Ireland) through the brain cannula (diameter 1.8 mm). Our practice is to use 3387 rather than 3389 electrodes (i.e. 1.5 mm rather than 0.5mm spacing between adjacent contacts), aiming to implant contacts within each of the following regions (ordered from ventral to dorsal); the substantia nigra reticula (for possible gait benefit and redundancy in case of a Z-axis positioning error), within the ventral and dorsal STN, and the region dorsal to that STN (for dyskinesia benefit) [37]. An intra-operative image intensifier confirmed macroelectrode depth and facilitated vertical adjustments to position the middle two contacts in the ventral and dorsal STN regions before electrode fixation to the skull (StimlocTM lead anchoring device, Medtronic Dublin, Ireland). Stimulation through the macroelectrode was not routinely assessed. The duration of surgery was available from data collected from a subgroup of 13 patients (26 hemispheres) operated at one centre. The mean duration, from return to theatre after the stereotactic CT (including the time taken to merge images, transfer the patient to the operating table and drape), until extubation after placement of the subcutaneous system, was 175.7 minutes (2.9 hours). A non-contrast CT was acquired within 24 hours after surgery (axial acquisition with 0.30–0.75 mm slice thickness), to assess electrode location via fusion with the preoperative MRI.

**Fig 1:**
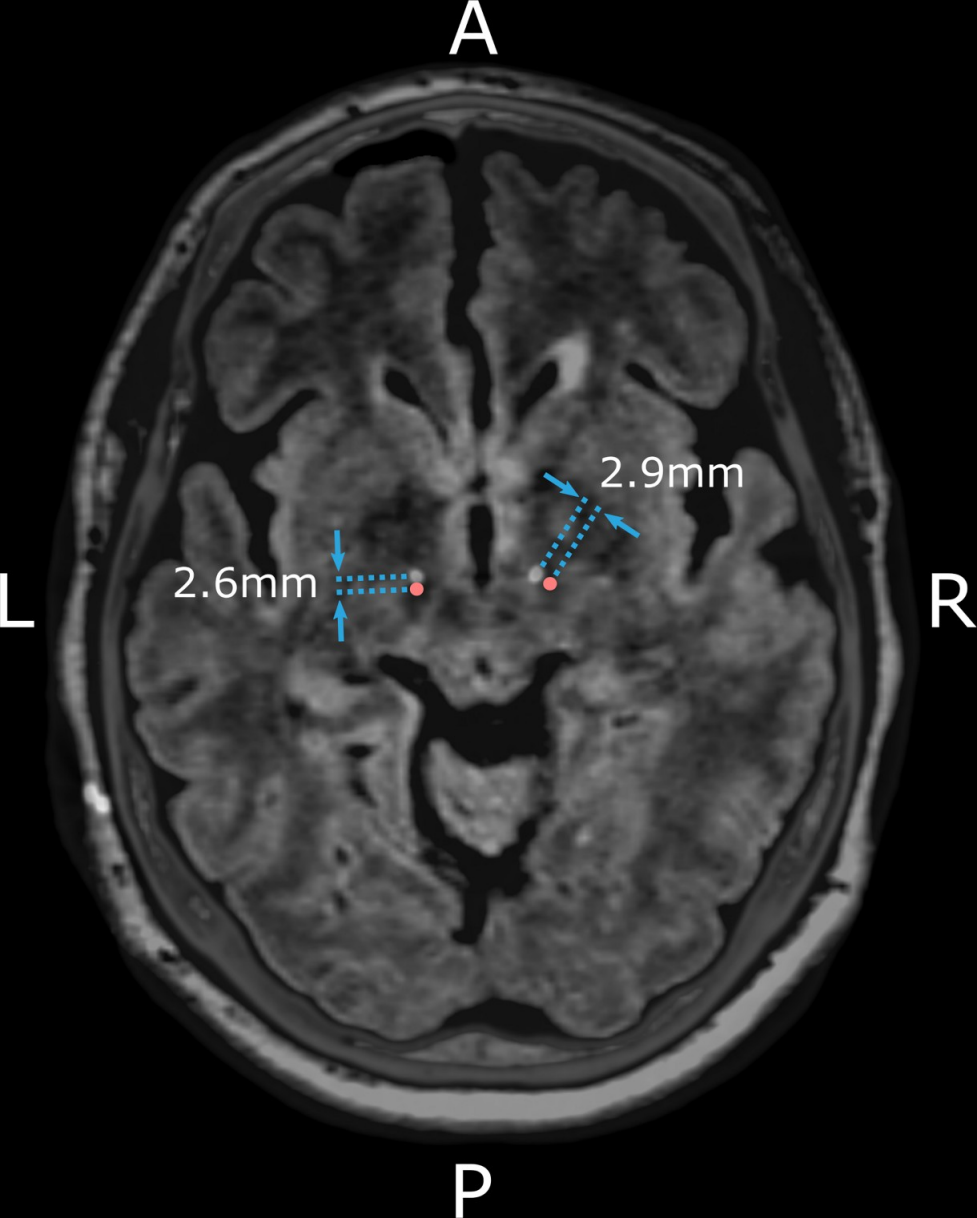
Assessing the error between the actual versus ideal STN-DBS electrode location. An example of the fused preoperative MRI and postoperative CT for a single patient with two electrodes implanted in the STN region. The nominated, ideal location to apply DBS in the dorsal STN is marked in red. The location of the electrode trajectory at this axial slice is defined by the electrode artefact occurring on CT (hyperintense voxels). The electrode location error is the straight-line (Euclidean) distance between the actual electrode trajectory versus the nominated, ideal location to apply DBS, as indicated by the blue arrows and measured in millimeters. A = Anterior, P = Posterior, L = Left, R = Right.

Patient imaging was anonymized with the order randomized. Using 3D Slicer (http://www.slicer.org, Harvard medical school, Boston, MA) [38], MRI images were aligned according to the anterior commissure–posterior commissure line and the midsagittal plane. A single point, nominated as the ideal location to apply DBS, was visually identified and manually marked in every hemisphere on native imaging (the preoperative FLAIR MRI), by an expert clinician (KB, Neurosurgeon) blinded to electrode location (Fig 1). This ideal location to apply DBS was calibrated between two expert clinicians (KB, Neurosurgeon and WT, Neurologist) in a subset of 30 MRI scans. The location, within the central area of the dorsal STN, 2mm below the superior margin of the adjacent red nucleus, was selected based on converging evidence suggesting that this subregion is, on average, the ideal location to apply DBS for motor benefit in PD [8, 20, 21]. Importantly, nomination of the ideal location involved marking a single point in each hemisphere without needing to find an appropriate trajectory to reach it.

Independently, on the postoperative CT, a research engineer (TP) marked the center of each (of four) contact artefacts and a line-of-best-fit determined the electrode trajectory. Each CT was inspected for intracerebral blood or ventricular transgression. The postoperative CT was then merged to the preoperative MRI. Image fusion was verified visually, and if deemed inadequate, the co-registration process was repeated with the aid of a mask. The mask enclosed a region-of-interest around the basal ganglia to prioritize fusion where electrode location was assessed [39]. Electrode location error was calculated as the Euclidean distance between the actual electrode trajectory and the nominated, ideal dorsal STN location, in the same axial plane, using a dedicated Python script (Python Software Foundation, version 3.7) [40].

The following information was retrieved from the operative notes; surgery date (yielding case number), first hemisphere implanted (left versus right), and number of microelectrode test trajectories per hemisphere. Shapiro-Wilk Normality tests were followed by either a Wilcoxon signed-rank test for paired samples or a Mann-Whitney U test for independent samples. A relationship between electrode location error and case number was sought using simple linear regression to seek a learning effect. Statistical differences were deemed significant if *p* < 0.05. All analysis was performed using Python software.

## Results

The 113 patients (72 male and 41 female) had a mean age of 60.7 (±7.9 SD) years, with data analysed for electrodes targeting 226 subthalamic nuclei. Intracerebral haemorrhage occurred in 3/226 (1.3%) hemispheres in 3 patients. All hemispheres with haemorrhage had been explored with only a single microelectrode pass. No electrodes traversed the ventricle.

**Fig 2:**
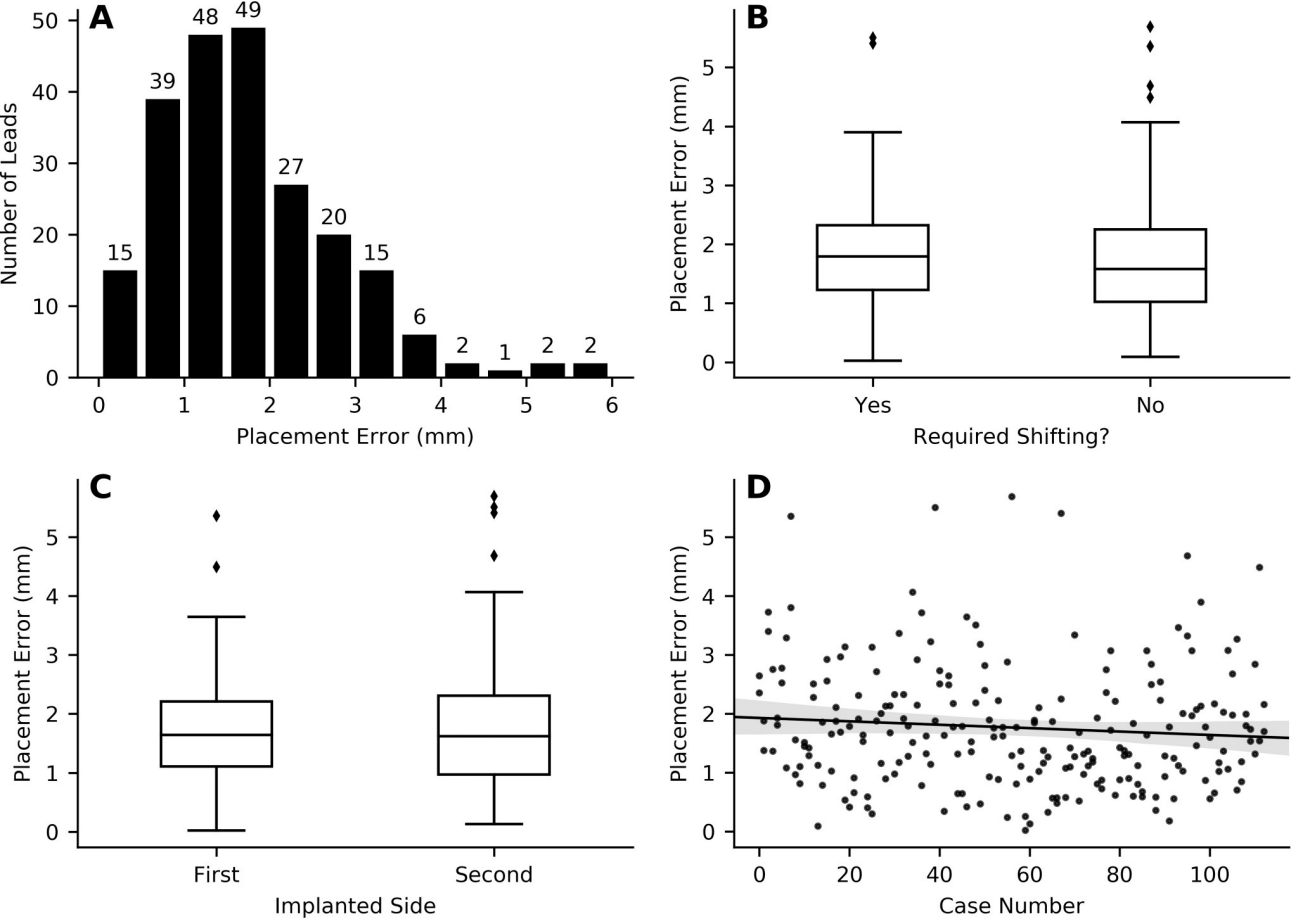
Results summary. The error in electrode placement between the nominated, ideal location to apply DBS in the dorsal STN versus actual electrode trajectory in 226 electrodes of 113 patients (A). An intraoperative microelectrode trajectory change was required in 48/226 (21.2%). There was no difference in electrode location error between electrodes preceded by one or more microelectrode trajectory changes versus those where no microelectrode trajectory change had occurred (Mann Whitney U-Test: U(47) = 3943, p = 0.21) (B). There was no difference in electrode location error between the first and second implanted side (Wilcoxon signed ranks: W(112) = 3106, p = 0.74) (C). There was no relationship between electrode location error and case number (linear regression: r = 0.09, p = 0.18, shaded region shows 95% confidence interval) (D).

The median electrode location error was 1.62 mm (IQR = 1.23). 151/226 electrodes (66.8%) were within 2 mm of the nominated, ideal location to apply DBS in the dorsal STN. 28/226 electrodes (12.4%) had an error exceeding 3 mm (Fig 2A). 8/226 (3.5%) electrodes in five patients were re-implanted to try and achieve a better location and the median initial error of these was 2.92 mm (IQR = 1.23) [41]. Of the reimplanted electrodes, 6/8 had been explored with only a single microelectrode trajectory at the first procedure. The clinical indication to reimplant electrodes in four patients was DBS induced gait and balance impairment and in one patient was insufficient tremor suppression.

A microelectrode trajectory change during surgery was required in 48/226 (21.2%) hemispheres. In these cases, the number of trajectories that were explored before the permanent electrode was implanted was as follows; two trajectories in 30 hemispheres, three trajectories in 17 hemispheres and four trajectories in one hemisphere. There was no difference in electrode location error between electrodes preceded by one or more microelectrode trajectory changes versus those where no microelectrode trajectory change had occurred (Mann Whitney U-Test: U(47) = 3943, p = 0.21, median ± IQR = 1.79 ± 1.09 vs 1.58 ± 1.22 mm, Fig 2B).

The left hemisphere was implanted first in 74/113 (65.5%) patients. There was no difference in electrode location error between the first and second implanted side (Wilcoxon signed ranks: W(112) = 3106, p = 0.74, median ± IQR = 1.64 ± 1.10 vs 1.62 ± 1.34 mm, Fig 2C). There was no difference in the frequency of microelectrode trajectory changes between the first and second implanted side (Wilcoxon signed-ranks: W(112) = 354.5, p = 0.44, median ± IQR = 1.0 ± 0.0 vs 1.0 ± 0.0).

There was no relationship between electrode location error and case number (linear regression: r = 0.09, p = 0.18, Fig 2D). There was no relationship between case number and the frequency of microelectrode trajectory changes (linear regression: r = -0.04, p = 0.59).

## Discussion

Here, in a consecutive cohort of 113 patients with PD implanted with STN-DBS by a newly established DBS team using awake microelectrode guided surgery, we assessed the error between the actual electrode trajectory versus a nominated, ideal location to apply DBS in the dorsal STN. The average error was 1.62 mm. However, there was substantial variability, with 12.4% of electrodes having an error > 3 mm. All procedures were bilateral and there was no difference in electrode location error between hemispheres implanted first or second. There was no relationship between electrode location error and case order, arguing against a learning effect. 21.2% of hemispheres required at least one microelectrode trajectory change before the permanent electrode was implanted. There was no difference in location error between electrodes positioned with or without a preceding trajectory change.

We first acknowledge several limitations of this research. The data arose from a single DBS surgical team working across hospitals in Melbourne, Australia. Results may not be generalizable to services employing different surgical techniques. However, we note that alternative implantation methods, such as the use of intra-operative imaging under general anaesthetic, have not been clearly demonstrated to yield better anatomical or clinical outcomes compared to the awake, microelectrode guided surgery that we assessed here [42]. Clinical details such as date of surgery, first and second operated hemisphere and occurrence of microelectrode trajectory changes were obtained retrospectively from medical records. However, these data were contemporaneously recorded and only one patient was excluded due to missing clinical data. We consider it unlikely that incorrect entry of such basic information would be a substantial confound. In contrast, the primary dataset of preoperative and postoperative imaging, was analysed after retrieval of the source data. Our primary endpoint was the error between the actual electrode trajectory versus an ideal location. The nature of this error could have been further characterized by also assessing the error between the actual versus intended trajectory (potentially allowing planning error and surgical implantation error to be discriminated). However, as we did not keep the surgical plans for the cohort, this analysis could not be performed. It is also important to recognise that this study aimed to assess electrode location only. The therapeutic effect of electrode location on motor outcomes and quality of life was not evaluated. Such an analysis would be confounded by other aspects of the clinical application such as patient selection and programming. Further limitations are also discussed below.

How does this study differ from previous studies that have assessed the error of DBS electrode implantation? Previous studies have assessed the discrepancy between the final electrode location versus the *planned* trajectory–which is an excellent measure of the technical constraints of stereotactic neurosurgery but does not account for limitations in the planning itself. Here, we assessed the proximity between electrode position versus the ideal location to apply DBS in the dorsal STN. This is a major determinant of therapeutic efficacy [9, 24]. This measure depends not only on errors that accrue during surgery but also how the trajectory is planned [6, 7, 10–12]. Importantly, in this study, the ideal location to apply STN-DBS was nominated as a single point whereas in clinical practice, a trajectory is also required to reach it. Trajectory plans need to accommodate for individual differences in anatomy such as cortical shape, ventricular size, blood vessel location and target orientation [13–15]. Moreover, trajectories can traverse the STN in different ways–ideally with some ‘redundancy’, so that if the dorsal ‘sweet spot’ is missed, that a more ventral zone with an acceptable therapeutic window is reached. For example, the clinicians in this study typically aimed to traverse the dorsal STN with an endpoint at the more ventral ‘Bejjani line’ [16]. What is clear, is that many different trajectories to the STN target can be taken in the same brain, and ultimately planning involves heuristic decisions that vary between, and even within, clinicians [17]. Such planning is a crucial skill in stereotactic neurosurgery and capturing this aspect of the clinical application of DBS is a key advantage of our method.

Of course, our method relies upon a valid definition and determination of the ideal location to apply STN-DBS. The average location of this ‘sweet spot’ is thought to lie in the dorsal STN, around 2 mm below the superior margin of the adjacent red nucleus [8, 9, 19–22]. After training and calibration, an expert clinician visually marked this point on FLAIR MRI, in every hemisphere. Variation in the manual marking of this location is a potential confound. However, such direct assessment on native imaging avoids the confounds inherent in referencing to a distant landmark such as the midcommissural point or distorting scans into a common imaging space [43]. In this study, we made the pragmatic assumption that the dorsal STN, identified on structural anatomy, identifies the ideal location to apply DBS in every patient. However, connectivity profiles of effective DBS suggest a variable relationship between the anatomical location of the STN and the functional pathways that need to be modulated for clinical benefit [9, 22, 44]. Our study was limited by the fact that we did not examine how electrode location in each patient affected the therapeutic outcomes from DBS. Tools to reliably localise these functional pathways, such as using advanced imaging or physiological techniques, are under development [8, 9, 45].

Whilst acknowledging these limitations, our method of assessing anatomical error does offer some important insights. The average error between actual versus ideal electrode location of 1.62 mm is not dissimilar to the range of errors previously reported for actual versus intended trajectories [4, 5, 46]. This average error would likely permit typical DBS parameters to reach and modulate the ideal location to apply STN-DBS. However, this error varied greatly, with 12.4% of electrodes positioned over 3 mm from the nominated, ideal location to apply DBS in the dorsal STN. Of course, the greater the error, the smaller the likely therapeutic window [47–49]. Our results therefore support the availability of steering electrodes, which may reduce the impact of electrode misplacement [50, 51]. However, these findings also suggest that despite steering electrodes, a proportion of patients may need surgery to reimplant electrodes to a better location [52]. There is emerging evidence highlighting the gravity of this issue, For example, a recent population based study of 1849 PD patients with DBS, found that 11% needed repeat surgery of the intracranial electrode [53]. We caution though, that our method does not account for the possibility that electrodes may miss the ideal location to apply DBS in the dorsal STN yet still encounter a clinically effective DBS zone elsewhere in the trajectory, especially ventrally. Acceptable outcomes from STN-DBS could result from proximity to such a zone rather than a specific point in the dorsal STN. Indeed, in our series, 8/226 (3.5%) electrodes were removed and replaced due to malposition–but there were many electrodes with greater measured error that were not reimplanted. However, many factors are involved in the decision to offer electrode revision surgery. For example, patients with partial benefit may be unwilling to undergo another intracranial procedure and clinicians may be reluctant to operate on frailer patients [54]. Clearly, better systems to navigate the implantation of STN-DBS electrodes are urgently needed. This appears to be an unmet need across surgical techniques, regardless of whether patients are awake or asleep or whether microelectrode recordings and intraoperative imaging are employed [18, 32].

The need for better methods to guide STN electrode implantation is also supported by the lack of remediable factors identified in this study that could have improved outcomes. For example, we found no difference in electrode location error between first and second implanted hemispheres. A difference in electrode location error between first and second implanted hemispheres has been variably reported in some series and attributed to brain shift during surgery [4, 27, 36]. Brain shift in this study may have been minimized by avoidance of the ventricle, short procedure duration, appropriate patient positioning, and use of dural sealant [10, 36]. We also found no relationship between electrode location error and case order. Thus, there was no evidence that greater surgical experience would have made a difference. Although our dataset included the commencement of a new DBS service, both members of the surgical team had substantial prior experience in DBS and were operating a high-volume service, factors which may improve surgical outcomes [29–31]. We also found no difference in location error between electrodes that were placed with (21.2%) or without a preceding microelectrode trajectory change. As we did not have intraoperative imaging, the exact anatomical path taken by each microelectrode trajectory is unknown. However, the most plausible explanation is that microelectrode trajectory changes reduced the error of errant trajectories towards the average (less likely is that shifts had occurred in better placed trajectories and increased the error towards the average). If the former is the correct interpretation, then this finding supports that, in the right setting, a microelectrode trajectory change will improve the final electrode location [55]. This is an important finding of this study, as it has been controversial as to whether microelectrode recordings and awake surgery with intraoperative clinical assessments can improve outcomes [18, 32, 33]. A potential risk of shifting microelectrode trajectories is intracerebral haemorrhage [18]. The rate of radiologically defined haemorrhage in this study, 3/226 hemispheres in 3 patients, was similar to many previous reports [56–59]. Interestingly, all three intracerebral haemorrhages occurred where no trajectory change had occurred. The distribution of electrode location errors could suggest that changing trajectory more often may have been beneficial. But this would require a more reliable system to determine actual versus ideal electrode location *during* STN-DBS surgery. One potential method is to use a physiological biomarker that indicates the ideal *functional* STN-DBS location with high location specificity, available intraoperatively [8, 60]. There is an urgent need to develop such methodology, as this study has demonstrated that the actual location of electrodes implanted even by experienced clinicians is highly variable, yet the outcomes of STN-DBS are known to be exquisitely dependent on proximity to the ideal stimulation location.

## Conclusion

The proximity of DBS electrodes to a nominated ideal location to apply DBS in the dorsal STN is highly variable, even when implanted by experienced clinicians with brain shift minimized, and without evidence of a learning effect. Using this measure, we found that assessments on awake patients (microelectrode recordings and clinical examination) likely yielded beneficial intraoperative decisions to improve positioning. Measuring the proximity of electrodes to the *ideal* rather than the *planned* location, captures the error arising from the planned trajectory in addition to the subsequent stereotactic process of implantation. In many patients in this study, this error is likely to have reduced therapeutic efficacy. Methods that allow a closer approximation of DBS electrodes to the ideal location to apply DBS in the dorsal STN are needed.

## Supporting information

S1 Dataset Study data. (XLSX)
